# Gintonin Isolated from Ginseng Inhibits the Epithelial—Mesenchymal Transition Induced by TGF-β in A549 Lung Cancer Cells

**DOI:** 10.3390/plants12102013

**Published:** 2023-05-17

**Authors:** Sung Jin Kim, Seung-Yeol Nah, Il-Ho Park, Myoung-Sook Shin, Ki Sung Kang

**Affiliations:** 1 College of Korean Medicine, Gachon University, Seongnam 13120, Republic of Korea; 2 Ginsentology Research Laboratory and Department of Physiology, College of Veterinary Medicine, Konkuk University, Seoul 05029, Republic of Korea; 3 College of Pharmacy, Sahmyook University, Seoul 01795, Republic of Korea

**Keywords:** epithelial-to-mesenchymal transition, gintonin, anti-metastatic activity, lung cancer

## Abstract

Epithelial-to-mesenchymal transition (EM transition) is a process wherein epithelial cells lose their intrinsic characteristics and cell–cell junctions and differentiate into a mesenchymal phenotype. EM transition is an important feature of cancer invasion and metastasis. In this study, we aimed to investigate the inhibitory effect of gintonin (GT), an ingredient of ginseng, on EM transition using A549 cells. The proliferation of A549 cells was enhanced following treatment with 50, 75, and 100 μg/mL of GT. GT affected EM transition-induced gene and protein expression, specifically that of vimentin (Vim), N-cadherin (N-cad), zinc finger E-box-binding homeobox 1, and Twist in A549 cells. Furthermore, the transforming growth factor beta 1 (TGF-β1)-induced phosphorylation of Smad2 and Smad3 was suppressed by GT treatment. Immunofluorescence staining also showed that GT treatment decreased the TGF-β1-induced expression of Vim and N-cad in A549 cells. Therefore, GT may be used to suppress cancer cell metastasis via maintenance of the cell–cell junction’s integrity. However, further studies are required to pave the way for its translation into clinical application in cancer therapeutics.

## 1. Introduction

Lung cancer is a devastating disease and one of the leading causes of cancer-related deaths worldwide [1]. Lung cancer is highly invasive and has the ability to metastasize by invading other tissues and spreading throughout the body [2]. Metastasis is a complex process that involves a series of molecular events, including the epithelial–mesenchymal transition (EM transition), which plays a crucial role in tumor invasion and dissemination [3]. During the EM transition, the intrinsic characteristics and cell–cell junctions of epithelial cells are lost, causing them to differentiate into a mesenchymal phenotype, which ultimately increases the invasion and migration capabilities of cancer cells [4]. A major characteristic of EM transition is the decrease in the levels of proteins involved in tight junctions, such as E-cadherin (E-cad), claudin, and occludin, which are epithelial markers. In contrast, there is an increased expression of mesenchymal markers, such as N-cadherin (N-cad), vimentin (Vim), and matrix metalloproteinases, during EM transition, indicating the acquisition of mesenchymal characteristics by these cells [3]. E-cad maintains the integrity of intercellular junctions, thus preventing cell migration, invasion, and metastasis and suppressing tumor progression. Gene expression of E-cad is reduced in most cancer cells [5] and regulated by E-cad transcriptional repressors Snail-1/2, zinc finger E-box-binding homeobox 1 (ZEB1)-1/2, and Twist. Snail, a zinc finger transcription factor, induces EM transition by suppressing the expression of E-cad [6]. Another important regulator of cancer development is transforming growth factor beta 1 (TGF-β1). The effects of TGF-β1 on tumor progression can be either suppressive or promotive, depending on the cancer stage [7]. In the early stages of cancer, TGF-β1 inhibits tumor growth by inducing cell cycle arrest and apoptosis; however, in the later stages, when tumor growth has reached or surpassed a certain size, cancer cells become resistant to growth suppression by TGF-β1. Therefore, TGF-β1 then induces EM transition and contributes to tumor growth and metastasis, angiogenesis, and immune evasion mechanisms, thereby promoting cancer progression [8,9].

*Panax Ginseng* C.A. Meyer is a natural product that is extensively used in Korea, China, and Japan [10]. It is composed of major saponins and non-saponins, with ginsenosides being a major component of the saponin group, whereas polysaccharides, proteins, peptides, and gintonins (GTs) belong to the non-saponin group [11]. Among them, GT is a glycolipoprotein fraction of ginseng and is composed of carbohydrates as the main ingredient, lipids, and proteins containing several hydrophobic and acidic amino acids, as well as glucose [12]. The major active of GT are lysophospholipids, including lysophosphatidic acids (LPAs), the receptor of which serves as a specific target for GT [13,14,15,16]. GT exhibits various health benefits, such as anti-Alzheimer’s disease effects, through the LPA receptor-mediated non-amyloidogenic pathway [17]. GT also improves cognitive function in older patients with Alzheimer’s disease [18,19], increases hippocampal neurogenesis [20], possesses anti-depressant activity [21], exerts in vivo anti-metastatic effects [22], and protects against cardiovascular diseases [23]. However, to the best of our knowledge, detailed studies on the inhibition of the intracellular signaling pathway of cancer metastasis by GT have yet to be performed. Therefore, in this study, we investigated, for the first time, the mechanism of action of GT in EM transition using A549 lung cancer cells.

## 2. Results and Discussion

### 2.1. Effect of GT on A549 Cell Proliferation

**Figure 1:**
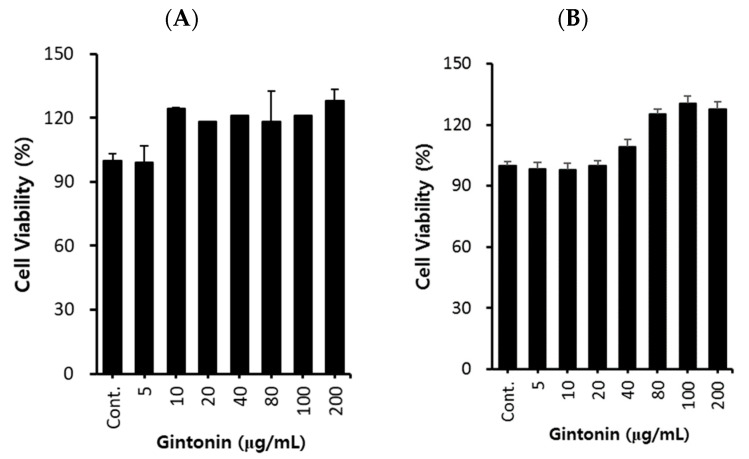
Effects of gintonin (GT) on A549 cell viability. Cells (2.5 × 10^4^ cells/well) were seeded in 96-well plates and then treated with various concentrations (5, 10, 20, 40, 80, 100, and 200 μg/mL) of GT for 24 (**A**) and 48 h (**B**). Cell viability was determined using an EZ-Cytox cell viability assay kit. Data are presented as the mean ± standard deviation (SD) of three independent experiments.

First, we investigated the cytotoxicity of GT on A549 lung cancer cells. After treating A549 cells with GT at concentrations of 5, 10, 20, 40, 80, 100, and 200 μg/mL for 24 and 48 h, cell viability was evaluated using the EZ-Cytox method. As shown in Figure 1A,B, cell proliferation tended to increase as the GT concentration increased without cell toxicity in A549 cells. Therefore, subsequent experiments were conducted within the above concentration range.

### 2.2. GT Suppressed the Expression of Transcription Factors Associated with TGF-β1-Induced EM Transition in A549 Cells

Epidermal growth factor, hepatocyte growth factor, bone morphogenetic proteins, and TGF-β1 are signaling pathway inducers that are well-known for their ability to activate EM transition-inducing transcription factors [7,8]. Among these, TGF-β1 is the most extensively studied EM transition-inducing protein [10]. Additionally, the Smad signaling pathway is a key mediator of the EM transition, and TGF-β1 plays a critical role in regulating this pathway. Upon TGF-β1 activation, it binds to TGF-βR1 and forms a complex that activates the Smad signaling pathway by phosphorylating Smad2 and Smad3 [24,25]. This activation regulates EMT transcription, contributing to the acquisition of mesenchymal properties [24,26]. Additionally, TGF-β acts on extracellular signal-regulated kinase (ERK), phosphoinositide 3-kinase, and p38, which affect the differentiation stages of cells undergoing EM transition [27].

**Figure 2:**
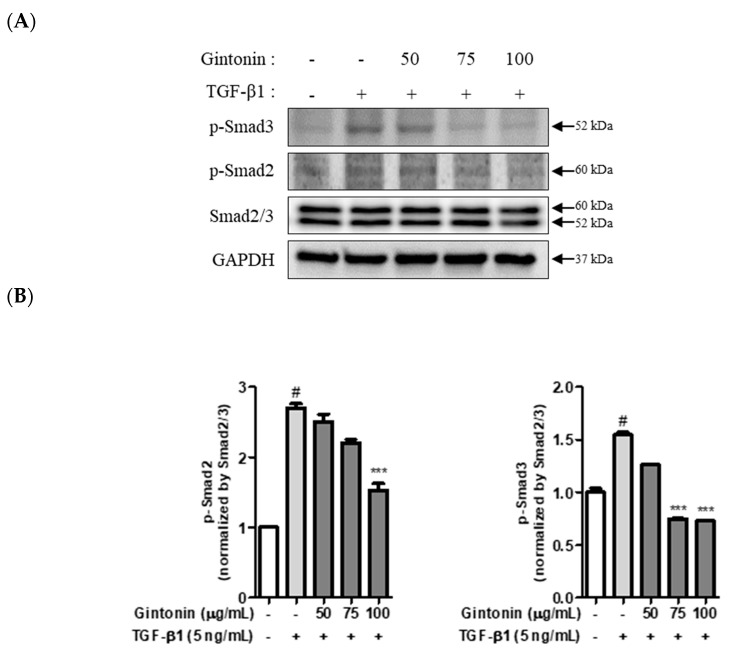

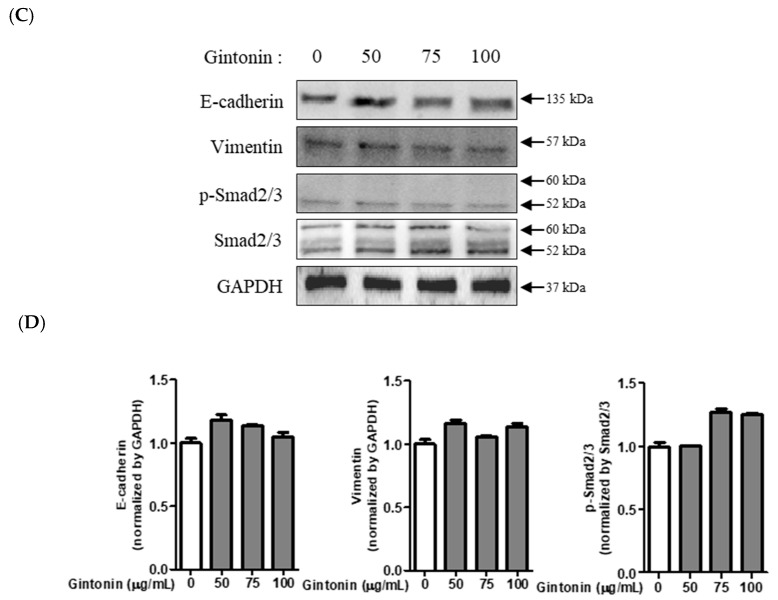
Gintonin (GT) inhibits Smad2/3 phosphorylation in transforming growth factor beta 1 (TGF-β1)-treated A549 cells. A549 cells (5 × 10^5^ cell/well, 6-well plate) were incubated overnight, and the medium was changed to serum-free RPMI 1640 to synchronize the cells. After 6 h, cells were pre-treated with GT (50, 75, and 100 μg/mL) for 4 h and then stimulated with TGF-β1 (5 ng/mL) for 30 min (**A**). A549 cells were treated with GT for 48 h (**C**). Cell lysates were analyzed via Western blotting using specific antibodies for E-cad, Vim, pSmad2, pSmad3, and pSmad2/3. Protein expression was quantified using Image J software (**B**,**D**). All data are presented as mean ± standard deviation (*n* = 3). # *p* < 0.0001 compared to the control group. *** *p* < 0.0001 compared to the TGF-β1 group.

Therefore, we examined the effect of GT on the phosphorylation of Smad2 and Smad3, which are downstream of the TGF-β1 receptor-related signaling pathway. A549 cells were treated with 50, 75, and 100 μg/mL of GT for 4 h, then incubated with TGF-β1 for 30 min. As shown in Figure 2A, Smad3 and Smad2 phosphorylation decreased in a concentration-dependent manner after GT treatment in A549 cells. In addition, we analyzed whether GT affected the phosphorylation of Smad2/3 in A549 cells. As shown in Figure 2C, GT treatment did not affect Smad2/3 phosphorylation in A549 cells. Moreover, the expression of epithelial cell marker proteins (E-cad) and mesenchymal cell marker proteins (Vim) did not change with GT treatment for 48 h in A549 cells. Based on these results, we speculated that the inhibition of Smad2 and Smad3 phosphorylation by GT treatment may affect the TGF-β1-induced signaling pathways, such as EM transition, in A549 lung cancer cells.

### 2.3. GT Suppressed the Expression of TGF-β1-Induced N-cad, Vim, and ZEB1 in A549 Cells

EM transition is an essential cellular process for embryogenesis, wound healing, fibrosis, and cancer progression [28]. During metastasis, TGF-β1 signals induce EM transition by activating various intracellular signaling pathways [29,30]. EM transition causes the destabilization of epithelial junction proteins, such as E-cad, occludin, and claudin, resulting in their cleavage and subsequent degradation in the plasma membrane [3,28,30]. This leads to a loss of cell-to-cell adhesion. Additionally, E-cad expression decreased, whereas N-cadherin expression increased, during EM transition. Furthermore, the expression of Vim, an intermediate protein filament in the cytoplasm of cells, increased [28]. Vim is responsible for maintaining the cytoskeleton and tissue structure of cells. EM transition involves several transcription factors, including ZEB1 and Twist. ZEB1, one of the regulators of cancer progression, downregulates E-cad gene expression and disrupts the adhesive junctions between epithelial cells [22,24].

**Figure 3:**
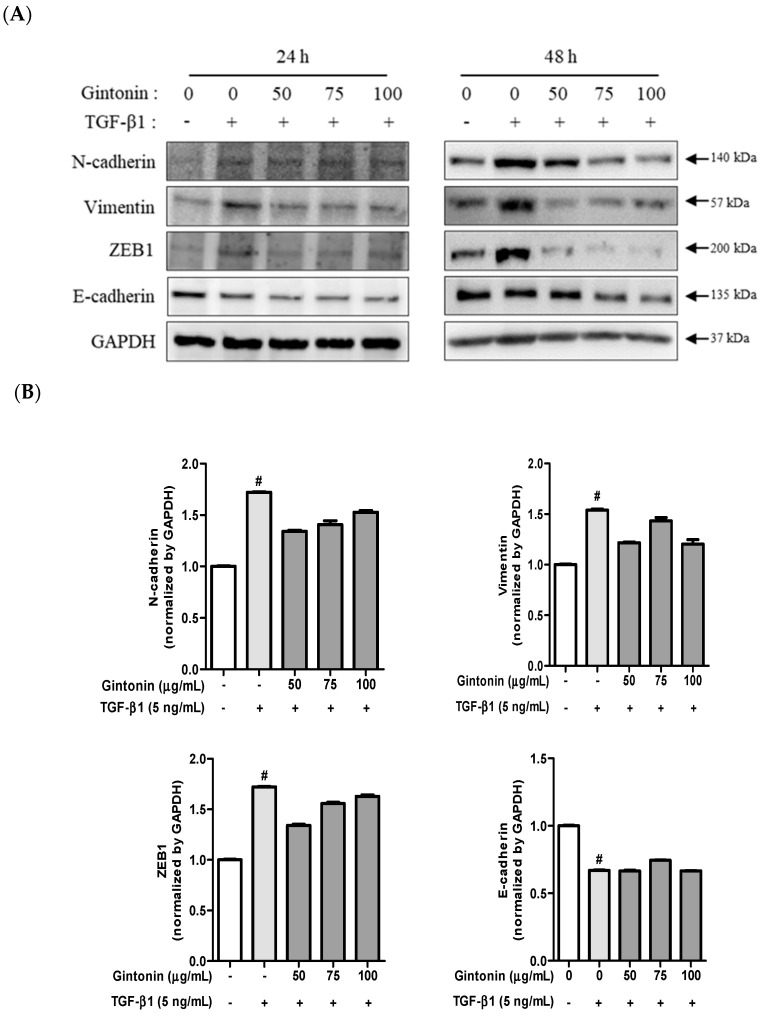

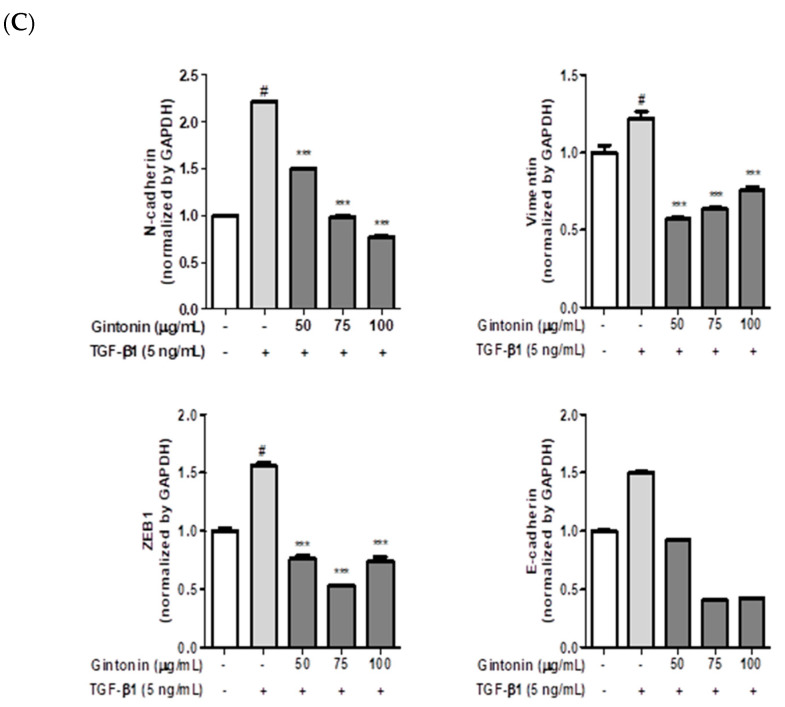
Gintonin (GT) suppresses the expression of transforming growth factor beta 1 (TGF-β1)-induced EM transition markers. A549 cells (5 × 10^5^ cells/well, six-well plate) were incubated overnight, then transferred to serum-free RPMI to synchronize the cells. Next, cells were treated with 50, 75, and 100 µg/mL of GT for 6 h and then incubated with TGF-β1 (5 ng/mL) for 24 and 48 h (**A**). The cell lysates were analyzed using Western blotting and identified using specific antibodies for N-cad, Vim, and ZEB1. Protein expression at 24 h (**B**) and 48 h (**C**) was quantified using ImageJ software. All data are presented as means ± standard deviation (*n* = 3). # *p* < 0.0001 compared to the control group. *** *p* < 0.0001 compared to the TGF-β1 group.

To investigate whether GT inhibits EM transition in A549 cells, cells were pretreated with GT for 6 h and EM transition was induced by stimulating the cells with TGF-β1 for 24 or 48 h. As shown in Figure 3A, the mesenchymal cell markers N-cad and Vim, as well as ZEB1 expression, slightly increased by TGF-β1 treatment for 24 h, and pretreatment of GT substantially decreased the expression of the TGF-β1-induced mesenchymal cell marker. While the epithelial cell marker E-cad’s expression markedly decreased by TGF-β1 treatment, GT treatment did not recover the expression of E-cad in A549 cells (Figure 3A, lift panel). When A549 cells were treated with TGF-β1 for 48 h, N-cad, Vim, and ZEB1 expression were strongly induced. However, when the cells were pre-treated with 50, 75, and 100 μg/mL of GT for 4 h, the expression of N-cad, Vim, and ZEB1 strongly decreased in a concentration-dependent manner. In addition, E-cad expression slightly decreased due to TGF-β1 treatment for 48 h, whereas GT did not recover E-cad expression (Figure 3A, right panel). Based on these results, it can be confirmed that GT has anti-metastatic potential, as it suppresses EM transition in lung cancer cells primarily by suppressing the expression of mesenchymal markers. However, GT was unable to affect TGF-β1-induced downregulated E-cad expression.

### 2.4. GT Suppressed the mRNA Expression of TGF-β1-Induced Mesenchymal Markers in A549 Cells

**Figure 4:**
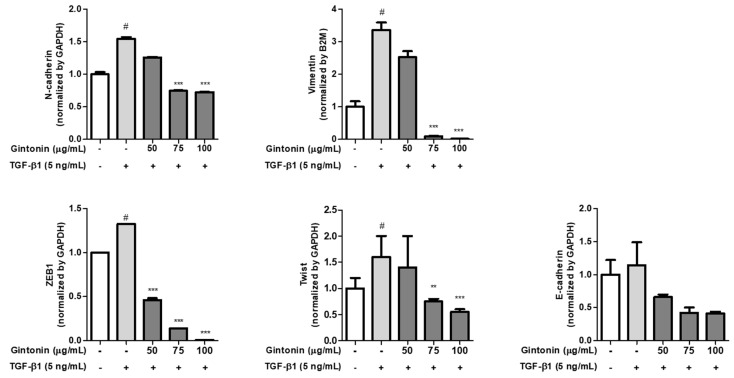
GT inhibited transforming growth factor beta 1 (TGF-β1)-induced increase in the mRNA levels during EM transition. A549 cells (5 × 10^5^ cell/well, six-well plate) were incubated overnight, and the medium was changed to serum-free RPMI 1640 to synchronize the cells. Next, cells were pre-treated with GT (50, 75, and 100 μg/mL) for 6 h, and then stimulated with TGF-β1 (5 ng/mL) for 18 h. mRNA expression was determined using qPCR. # *p* < 0.0001 compared to the control group, and *** *p* < 0.0001 and ** *p* < 0.001 compared to the TGF-β1 group.

Next, we investigated the effect of GT on EM transition-related gene expression by analyzing the expression of four representative mesenchymal genes, including N-cad, Vim, ZEB1, and Twist. A549 cells were treated with GT for 4 h and then with TGF-β1 for 18 h. As shown in Figure 4, mRNA expression was significantly induced by TGF-β1 treatment. While the upregulated mRNA expression of N-cad, Vim, ZEB1, and Twist decreased as the concentration of GT increased, the mRNA expression of E-cad increased with GT treatment; however, there was no statistical significance. Therefore, it can be predicted that GT treatment does not directly affect E-cad expression. In addition, the mRNA expression results correlated with the protein expression results, as shown in Figure 3A. Based on the results shown in Figure 4, we confirmed that GT inhibited EM transition by regulating the mRNA expression of N-cad, Vim, ZEB1, and Twist, which are essential in EM transition.

### 2.5. Effect of GT on Morphological Changes and Mesenchymal Cell Markers in TGF-β1-Induced EM Transition

**Figure 5:**
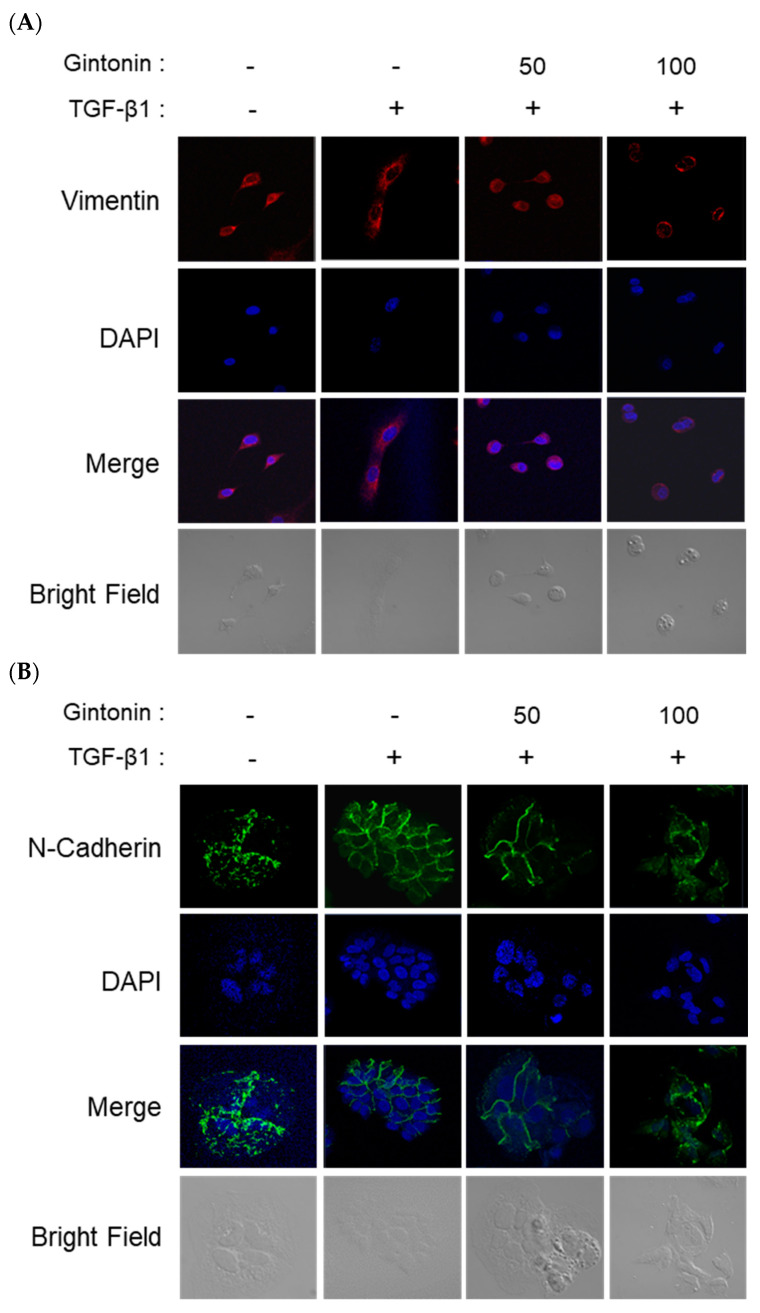
Gintonin (GT) suppressed the expression of vimentin and N-cadherin in transforming growth factor beta 1 (TGF-β1)-induced EM transition. A549 cells were seeded on a coverslip and incubated with RPMI 1640 medium. Thereafter, the medium was changed to serum-free RPMI 1640 medium for 6 h. Cells were pre-treated with GT (50 μg/mL and 100 μg/mL) for 4 h, and then stimulated with TGF-β1 (5 ng/mL) for 42 h. Immunofluorescence staining was performed as described in the Materials and Methods section to visualize vimentin and N-cadherin expression using confocal microscopy. Red and green fluorescence indicate the expression of vimentin (**A**) and N-cadherin (**B**), respectively. The nuclei were stained using DAPI.

As previously alluded to, morphological changes that occur during the EM transition process enable cancer progression [7,25,28]. Therefore, we observed the inhibition of EM transition by GT using confocal microscopy. As depicted in the bright field of Figure 5A,B, control cells (not treated with TGF-β1) had an epithelial appearance and adhered to each other. However, TGF-β1-treated cells became mesenchymal, and were elongated and spindle-shaped. Immunofluorescence staining of Vim (red dye) showed that GT treatment inhibited TGF-β1-induced Vim expression in a concentration-dependent manner. In addition, GT-treated cells displayed an epithelial morphology compared with those in the TGF-β1 group (Figure 5A). Similar results were observed following N-cad immunofluorescence staining (Figure 5B). The expression of N-cad (green dye) increased with TGF-β1 treatment, whereas GT treatment strongly inhibited N-cad expression in A549 cells. These results correlate with those shown in Figure 2. The expression of Vim is induced by TGF-β1 activation, Snail expression, and ERK phosphorylation [26]. The suppression of Vim and N-cad not only reduces motility during cancer metastasis, but also partially restores the epithelial cell phenotype in numerous cancer cell lines [23,26]. Therefore, GT can successfully suppress EM transition, preventing cancer cell migration and invasion.

## 3. Materials and Methods

### 3.1. Antibodies and Reagents

Antibodies against N-cad, Vim, ZEB1, E-cad, Smad2, Smad3, phosphorylated Smad2, and phosphorylated Smad3 were sourced from Cell Signaling Technology (Danvers, MA, USA). RPMI 1640 medium and TGF-β1 were obtained from Gibco (Grand Island, NY, USA) and PeproTech (Rocky Hill, NJ, USA), respectively. Invitrogen (Carlsbad, CA, USA) provided Alexa Fluor 488-IgG antibody for Vim, Texas Red-IgG antibody for N-cad, and 4′,6-diamidino-2-phenylindole (DAPI) for nuclear staining. The eight-well glass chamber slide was obtained from Nunc (Nalge Nunc International, Rochester, NY, USA).

### 3.2. Preparation of GT from Ginseng

The GT-enriched fraction of P. ginseng was prepared following our previous protocols [14,23]. Four-year-old ginseng was extracted using ethanol (yield: 35%). Ethanol extraction and centrifugation were performed, followed by lyophilization of the precipitate to produce the GT-enriched fraction. Our previous study identified the GT-enriched fraction as containing fatty acids (7.53% linoleic acid, 2.82% palmitic acid, and 1.46% oleic acid), 0.6% lysophospholipids and phospholipids, and 1.75% phosphatidic acids, as determined using liquid chromatography–mass spectrometry/mass spectrometry [14,23,31]. The GT-enriched fraction was dissolved in dimethyl sulfoxide (DMSO) for further analysis and diluted with media before treatment in A549 cells.

### 3.3. Cell Culture

The human lung cancer cell line A549 was obtained from the Korean Cell Line Bank (KCLB, Seoul, Republic of Korea) and cultured in RPMI medium, supplemented with 10% fetal bovine serum and 1% penicillin–streptomycin, under standard conditions of 37 °C and 5% CO_2_.

### 3.4. A549 Cell Viability Analysis

To assess the effect of GT on A549 cell viability, we used the EZ-Cytox cell assay kit (DoGenbio, Seoul, Republic of Korea). Briefly, cells were seeded in a 96-well plate (2.5 × 10^4^ cells/well) and treated with different concentrations of GT for 24 or 48 h. GT was prepared in DMSO and diluted to 5–200 μg/mL in the medium. After incubation, EZ-Cytox solution was added to the cells and incubated for 1 h, and the absorbance was measured at 450 nm using a 96-well plate reader (Emax, Molecular Devices, San Jose, CA, USA).

### 3.5. Induction of EM Transition via TGF-β1 Treatment

A549 cells (5 × 10^5^ cells/well) were seeded in RPMI medium in six-well plates and incubated overnight. The medium was then replaced with serum-free RPMI medium containing 1% penicillin–streptomycin, and the cells were treated with different concentrations of GT (50, 75, and 100 μg/mL) for 4 h. Subsequently, the cells were incubated with TGF-β1 (5 ng/mL) for 48 h, as previously reported [32,33].

### 3.6. Immunoblotting

A549 cells (5 × 10^5^ cells/well) were cultured in six-well plates and treated with GT for 48 h to assess the expression of E-cad, Vim, p-Smad2/3, and Smad2/3. For phosphorylation analysis of Smad2 and Smad3, cells were treated with GT for 4 h before TGF-β1 exposure for 30 min. N-cad, Vim, ZEB1, and E-cad expression levels were evaluated after 48 h of GT treatment. Cell lysates were prepared in RIPA buffer (T&I, Chuncheon, Republic of Korea) supplemented with protease inhibitor, dithiothreitol, and phosphate inhibitor, followed by centrifugation and separation of protein samples using TGX gel electrophoresis. PVDF membranes were incubated overnight in 5% skim milk/TBS-T buffer, probed with primary antibodies, and then incubated with secondary antibodies. Protein bands were visualized and quantified using ImageJ software and normalized to GAPDH levels. Phosphorylation levels were determined by comparing the samples with their non-phosphorylated counterparts.

### 3.7. Real-Time Quantitative Reverse Transcription Polymerase Chain Reaction (RT-qPCR)

For mRNA expression analysis, A549 cells were seeded at a density of 5 × 10^5^ cells/well in a six-well plate and incubated overnight. After changing the medium to serum-free medium, cells were treated with GT at concentrations of 50, 75, and 100 μg/mL for 6 h, followed by treatment with TGF-β1 (5 ng/mL) for 18 h. Total RNA was extracted using an AccuPrep Universal RNA Extraction Kit (Bioneer, Daejeon, Republic of Korea) and reverse-transcribed using an AccuPower RT premix (Bioneer). Real-time PCR was performed using the TaqMan gene expression assay kit (Applied Biosystems, Foster City, CA, USA) or SYBR Green Master Mix (Applied Biosystems), with specific primers for E-cad (sense and anti-sense primers 5′-CCACCAAAGTCACGCTGAATAC-3′ and 5′ GAAGAAGAGGACAGCACTG-3′), Vim (Hs_00958111_m1), N-cad (Hs_00983056_m1), ZEB1 (Hs_01566408_m1), Twist (Hs00361186_m1), and GAPDH (Hs_02786624_m1). qPCR results were determined from triplicate reactions. mRNA levels were determined by the Quant 3 PCR system (Applied Biosystems). 

### 3.8. Immunofluorescence Staining for Confocal Microscopy

A549 cells (3.0 × 10^4^ cells/well) were cultured on an eight-well chamber slide and incubated overnight. After treatment with GT (50 and 100 g/mL) for 6 h, the cells were further incubated with TGF-β1 for 42 h. The cells were then fixed with 4% paraformaldehyde, permeabilized with 1% Triton X-100, and blocked with 1% normal goat serum. Texas red-conjugated Vim or Alexa Fluor 488-conjugated N-cad antibody treatment was conducted at room temperature, followed by DAPI staining. The expression of Vim and N-cad was observed using an LSM700 confocal microscope (Carl Zeiss, Oberkochen, Germany)

### 3.9. Statistical Analysis

All graphs were constructed using mean ± standard deviation results calculated from triplicate analyses. Data were analyzed via one-way analysis of variance followed by Tukey’s post-hoc test using GraphPad Prism 8 software (GraphPad, Inc., San Diego, CA, USA).

## 4. Conclusions

Cancer metastasis, which is characterized by the spread of cancer cells throughout the body to create secondary tumors, is responsible for 90% of cancer-related deaths [34]. Lung cancer, which is the most prevalent type of cancer worldwide, has the highest mortality rate among all cancers [35]. Despite recent advances in lung cancer diagnosis and surgical techniques and an increase in the efficiency of chemotherapy, the survival rate of patients with lung cancer has not changed. Therefore, recently, cancer treatment strategies using natural plant-derived ingredients have received considerable research attention.

**Figure 6:**
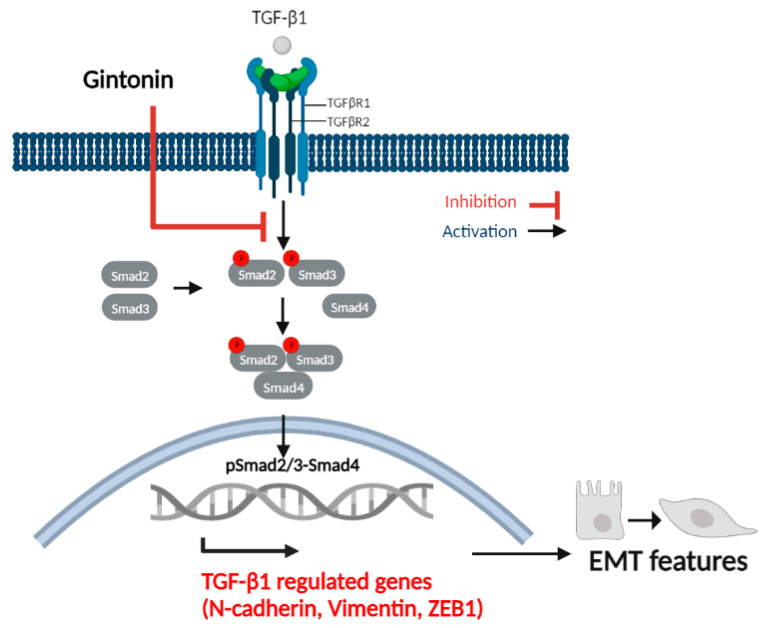
Inhibitory mechanism of GT on TGF-β1-induced EM transition in A549 cells.

GT activates LPA receptors to regulate various intracellular activities that suppress inflammation and perform various functions in cells, including those related to cell proliferation, migration, and vascular development. In the present study, we evaluated the inhibitory effect of GT on TGF-β1-induced EM transition in A549 cells and identified the regulated signal pathways. GT decreased protein expression (Vim, N-cad, and ZEB1) and mRNA levels (Vim, N-cad, ZEB1, and Twist) in A549 cells. In addition, GT substantially suppressed TGF-β1-regulated Smad-dependent signaling, such as phosphorylation of Smad2 and Smad3 (Figure 6). Previous studies have demonstrated the inhibitory effects of epigallocatechin gallate (EGCG), geraniin, and sanguiin H6, which are found in *Camellia sinensis*, *Phyllanthus amarus*, and *Sanguisorbae Radix*, respectively, on TGF-β1-induced EM transition in lung cancer by targeting the Smad signaling pathway [36,37,38]. Although GT, a compound extracted from ginseng, has shown promising anticancer potential against various types of cancer in vitro, predicting its efficacy in vivo remains a challenge. Therefore, further extensive research and clinical studies are necessary to provide robust evidence of its anti-cancer mechanisms and clinical applications [39,40].

Conclusively, GT warrants further investigation as a potential therapeutic agent for inhibiting early metastasis in lung cancer.
